# Brain volumes in adults with congenital heart disease correlate with executive function abilities

**DOI:** 10.1007/s11682-020-00424-1

**Published:** 2021-01-30

**Authors:** Nadja Naef, Ladina Schlosser, Peter Brugger, Matthias Greutmann, Angela Oxenius, Flavia Wehrle, Raimund Kottke, Beatrice Latal, Ruth Tuura O’Gorman

**Affiliations:** 1 Child Development Center, University Children’s Hospital Zurich, Steinwiesstrasse 75;, CH –8032 Zurich, Switzerland; 2 Department of Neurology, University Hospital Zurich, Zurich, Switzerland; 3 Department of Cardiology, University Heart Center, University Hospital Zurich, Zurich, Switzerland; 4 Pediatric Cardiology, Pediatric Heart Center, University Children’s Hospital Zurich, Zurich, Switzerland; 5 Children’s Research Center, University Children’s Hospital Zurich, Zurich, Switzerland; 6 Department of Diagnostic Imaging, University Children’s Hospital Zurich, Zurich, Switzerland; 7 MR Research Center, University Children’s Hospital Zurich, Zurich, Switzerland

**Keywords:** ACHD, Congenital heart disease, Brain volume, Brain imaging, Executive function

## Abstract

Congenital heart disease is the most common birth defect, and patients are at risk for neurodevelopmental impairment and brain abnormalities. Yet, little is known about the link between brain volumes and cognitive function in adults with congenital heart disease. Forty-four patients and 53 controls between 18 and 32 years underwent brain magnetic resonance imaging and cognitive testing, assessed with an intelligence quotient and executive function global score. Associations between brain volumes and cognitive function were calculated using linear models. Cognitive function in patients was within the normal range (intelligence quotient: 97.74 (10.76)). Total brain volume was significantly smaller in patients compared to controls (1067.26 (113.53) vs 1113.04 (97.88) cm^3^, *P <* 0.01), irrespective of cardiac factors (heart defect complexity, cyanosis, cardiopulmonary bypass: all *P* > 0.4). After adjusting for total brain volume, only corpus callosum volume remained significantly smaller (*P =* 0.03). Smaller total brain volume was associated with poorer overall executive functioning (*P =* 0.02) and inhibition (*P* < 0.01), in both patients and controls. The association between total brain volume and overall executive functioning was moderated by parental socioeconomic status (lower socioeconomic status was associated with a stronger association between brain volume and EF; interaction *P* = 0.03). In adults with congenital heart disease, despite normal intelligence quotient, brain volume alterations persist into adulthood and are related to executive functioning, in particular inhibitory control. Adults coming from low socioeconomic background and with altered brain volumes are especially vulnerable and should thus be followed-up during adulthood to ensure optimal social and educational support.

**Supplementary Information:**

The online version contains supplementary material available at 10.1007/s11682-020-00424-1.

## Introduction

Congenital heart disease (CHD) is the most common birth defect, occurring in about 8/1000 live births (van der Linde et al. 2011). With recent advances in medical care, more than 90% of neonates born with complex CHD reach adulthood, and adults with CHD now outnumber children with CHD (Marelli et al. 2016). Children with CHD are at risk for neurodevelopmental morbidity, such as cognitive, language and motor impairment and behavioral problems (Latal 2016). Evidence suggests that cognitive difficulties persist into adulthood (Tyagi et al. 2014; Jackson et al. 2015).

Magnetic resonance brain imaging (MRI) in the CHD population has revealed a variety of structural and functional brain abnormalities, including altered brain volumes and connectivity in neonates, and perioperative white matter injuries (Hagmann et al. 2016; Peyvandi et al. 2019). These alterations seem to persist into adolescence and are associated with poorer neurocognitive functioning (Brossard-Racine et al. 2014; Rollins et al. 2014; Brewster et al. 2015; von Rhein et al. 2015; Hagmann et al. 2016; Rollins et al. 2017; Bolduc et al. 2018; Watson et al. 2018; Peyvandi et al. 2019; Kessler et al. 2020).

Neuroimaging studies in adults with CHD (ACHD) suggest that brain abnormalities persist into adulthood (Horigome et al. 2006; Heinrichs et al. 2014; Brewster et al. 2015; Melazzini et al. 2019). To date, only few studies have assessed brain volumes in ACHD (Cordina et al. 2014; Semmel et al. 2018; Fontes et al. 2019). One study showed that cerebellar volumes were smaller in ACHD (Semmel et al. 2018) Another study found an association between hippocampal volume reduction and poorer executive function (EF), however, EF were assessed by means of a questionnaire and not with direct, detailed assessment (Fontes et al. 2019). EF are complex, higher-order self-regulatory and control processes which become increasingly important in adolescence and adulthood and EF deficits have been linked to reduced brain volumes in adolescents with CHD (Bolduc et al. 2018). To our knowledge, no study has examined the relationship between IQ, EF and altered brain volumes in ACHD, using a standardized neuropsychological test battery.

Therefore, the purpose of the present study was twofold: 1) to investigate total brain volume (TBV), grey and white matter volume in a current population of ACHD in comparison to healthy controls, and 2) to explore the relationship between brain volumes, IQ and executive functioning, assessed with an extensive test battery.

## Methods

### Study design

In this cross-sectional study, participants underwent neuropsychological assessments at the Zurich University Hospital and magnetic resonance brain imaging (MRI) at the University Children’s Hospital Zurich.

### Study population

Young adults with CHD aged 18 to 32 years were recruited from two previous studies (von Rhein et al. 2011; Rometsch et al. 2018) between 2016 and 2018 at the University Hospital Zurich. Patients were recruited at the time of their clinic visit or by telephone. Exclusion criteria were comorbidities affecting cognitive functioning (i.e. genetic disorders, severe psychiatric disease and severe neurological impairments) or if patients were not fluent in the German language. Healthy controls matched to the ACHD with respect to age, sex and education were recruited as peers of the ACHD or through personal contacts or public posts.

### Medical and demographic variables

Demographic variables were collected through questionnaires: Schoolyears were measured as the number of schoolyears until the first graduation. Parental socioeconomic status (SES) was estimated based on parental educational levels, using a 12-point scale ranging from 2 (lowest) to 12 (highest) SES. Medical variables were collected from the medical records and included: medical diagnosis, cardiopulmonary bypass (CPB) time, number of surgeries using CPB and complexity of CHD (mild, moderate and severe) (Warnes et al. 2001).

### Cognitive function

Intelligence quotient (IQ) was estimated using a short form of the Wechsler Adult Intelligence Scale, Fourth Edition, (WAIS-IV) (Daseking et al. 2014).

EF was assessed using an extensive test battery to capture the multidimensional and overlapping nature of executive functions (Miyake and Friedman, 2012; Baggetta and Alexander 2016). The following tests were included: the color-word-interference test of the Delis-Kaplan Executive Function System (D-KEFS) (Delis et al. 2001) to assess inhibitory control and cognitive flexibility. The 5-Point Test (Regard et al. 1982) to evaluate nonverbal fluency. The S-word subtest of the Regensburger Wortflüssigkeitstest (RWT) (Aschenbrenner et al. 2000) to assess verbal fluency. Standardized Link’s Probe to assess goal-oriented action planning (Metzler 2000). Trail Making Test (TMT) to assess flexibility (Reitan and Wolfson 1985). The computer-based stop signal task STOP-IT (Verbruggen et al. 2008) was applied to measure response inhibition. The subtest verbal digit (Petermann 2012) and visual block span (WMS-R) (Härting 2000) were used to measure working memory. Test results were expressed as T-values according to the respective test manuals. From the T-values, an average was calculated to form a mean EF global score. Further, an average score for inhibition, working memory, flexibility, fluency, and planning was calculated.

### Brain magnetic resonance imaging

#### Image acquisition

Participants underwent a research cerebral MRI on a 3T GE MR750 scanner. Hearing protection was provided with earplugs and headphones. For volumetric analysis, high-resolution three-dimensional (3D) T1 weighted images were acquired, using a 3D spoiled gradient echo (SPGR) pulse sequence (scan parameters: repetition time/echo time (TR/TE) = 11/5 ms; inversion time = 600 ms; flip angle = 8°; acquisition matrix = 256 × 192, reconstruction matrix: 256 × 256; field of view (FOV) = 256 cm).

Structural brain abnormalities have been previously described by Kessler et al. 2020 (Kessler et al. 2020): MR images were assessed for abnormalities by an experienced neuroradiologist (R.K.). who was blinded to group status (ACHD versus controls). Brain abnormalities were classified into three categories: focal infarction or atrophy, white matter lesions and microhemorrhages (Kessler et al. 2020).

#### MRI processing

Cortical reconstruction and volumetric segmentation was performed with the Freesurfer image analysis suite version 5.3.0, which is documented and freely available for download online (http://surfer.nmr.mgh.harvard.edu/). For statistical analyses, volumes were pooled over both hemispheres. Cerebellar volume was calculated using cerebellar white matter due to decreased image contrast in the lower cerebellum.

### Statistics

Descriptive statistics include mean and standard deviation (SD) or median and interquartile range (IQR) and frequencies. Group differences were calculated using the t-test and for non-normal variables the Mann-Whitney-U test. The *χ*^2^ test was used for categorical variables.

Brain volumes were analysed in a top-down approach: First, we investigated whether TBV was different between groups. Second, we analysed which of the following substructures mainly accounted for the difference in TBV: total white matter, total gray matter, cerebellum and cerebrospinal fluid. In addition, we compared corpus callosum as a particularly affected structure in the CHD (Hagmann et al. 2016; Bolduc et al. 2018). TBV and substructures were compared between groups using separate linear regression models for each brain volume/substructure, with brain volume/substructure as dependent and group (ACHD vs control) as independent variable. Regression models were re-run for all brain substructures, which differed significantly between groups, including TBV as a covariate.

In ACHD, to assess the influence of medical risk factors (cyanosis, complexity of heart defect, use of CPB surgery) on TBV, univariate regression models were calculated for each medical factor separately, with TBV as dependent and medical risk factor as independent variable were calculated.

The association of brain volumes and neurocognitive function (EF and IQ) was calculated for TBV and those substructures which remained significantly different between groups, after adjusting for TBV, namely the corpus callosum. Univariate linear regression models were calculated including EF or IQ as dependent variable and group, brain volume and an interaction of group with brain as independent variables, adjusted for TBV.

The effect of SES on TBV was calculated using univariate linear regression with TBV as dependent and SES category, group and an interaction of group with SES as independent variables. Thereafter, a model with EF global score as dependent variable and SES, TBV and an interaction of SES with TBV was calculated for all subjects.

To adjust for multiple comparisons, a FDR (Benjamini-Hochberg-Method) correction was used (Cao and Zhang 2014). Regression models, were adjusted for sex and age. Corrected two-tailed *P*-values <0.05 were considered significant. Analysis was conducted with the R software. (R core team, version 3.4.2, URL https://www.Rproject.org/.).

## Results

### Study population

#### ACHD

Of 198 subjects identified from two previous studies (von Rhein et al. 2011; Rometsch et al. 2018), 7 were excluded due to Marfan syndrome and one subject due to cardiomyopathy. Of 190 eligible ACHD, 59 could not be contacted and 64 declined participation. Of 67 ACHD, all underwent neuropsychological assessment and 46 underwent brain imaging (MRI safety not ensured *n* = 12, claustrophobia *n* = 2, obesity *n* = 1, pregnancy *n* = 1, refused *n* = 5).

There was no difference in sex and disease complexity (sex: *P* = 0.69, disease complexity: *P =* 0.34) between participants and non-participants. Non-participants were younger (*P* < 0.001) and had more systemic ventricular dysfunction (ejection fraction below 52%) compared to participants (*P* = 0.03).

#### Controls

Of 55 controls, 54 underwent neuropsychological assessment and neuroimaging (refused MRI *n* = 1).

MRI processing with initial segmentation was successful in 97 subjects (ACHD: 44, controls: *n* = 53). Three subjects (ACHD: *n* = 2, control: *n* = 1) had insufficient image quality and were discarded from further analysis. Thus, we here report data of 44 ACHD and 53 controls with a complete neuropsychological assessment and successfully segmented brain volumes. Subject characteristics are presented in Table 1. ACHD and controls did not differ in sex, age and parental SES. For cardiac diagnosis of ACHD see supplemental Table 4.

**Table 1:** Subject characteristics of ACHD subjects and controls

	ACHD, *N* = 44	Controls, N = 53	*P*
Age at assessment (years), M (SD)	26.71 (3.58)	25.90 (3.30)	0.25
Female participants, N (%)	17 (38.6)	26 (49.1)	0.41
Parental SES, Median (IQR)^a^	8 (7.75; 10)	9 (8; 10)	0.09
School education (years), Median (IQR)	13.25 (12; 15)	15.00 (14; 16)	0.01
IQ, Mean (SD)	97.74 (10.76)	104.02 (12.15)	0.01
EF global score, Mean (SD)^b^	50.29 (5.43)	52.46 (4.13)	0.03
Inhibition	48.81 (5.82)	51.18 (6.95	0.08
Working memory	50.59 (6.25)	52.83 (6.63)	0.09
Flexibility	50.94 (7.73)	54.05 (6.31)	0.03
Planning^b^	49.15 (13.97)	49.96 (11.80)	0.76
Fluency	50.44 (6.52)	52.61 (5.21)	0.07
Heart defect complexity, N (%)			
mild	15 (33.3)		
moderate	20 (45.5)		
severe	9 (20.5)		
Cardiopulmonary bypass surgery, N (%)	31 (68.9)		
Number of CPB^c^ surgeries, N (%)			
0	13 (29.5)		
1	21 (47.7)		
2	4 (9.1)		
3	6 (13.6)		
ECC time^d^ minutes, Median (IQR)	137 (65.25; 190.00)		
Age in years at first CPB^c^ surgery, Median (IQR)	1.18 (0.52; 8.10)		
Univentricular defect, N (%)	2 (0.5)		
Cyanotic heart defect, N (%)	13 (29.5)		
Cyanosis at time of assessment^e,^ N (%)	2 (0.05)		

^a^eight missing values; ^b^one missing value, ^c^eight missing values; ^d^11 missing values; ^e^SpO2 < 90% at assessment measured by pulse oximetry; SES: Socioeconomic status calculated from parental education; CPB: Cardiopulmonary bypass

Mean IQ and mean EF global score were significantly lower in ACHD compared to controls, but within the normal range in both groups (IQ: 97.74 (10.76) vs 104.02 (12.15), *P* = 0.01; EF: 50.18 (5.77) vs 52.37 (4.11), *P* = 0.03).

### Brain imaging

Brain abnormalities were observed in ACHD but not in controls: Eight (18.18%) ACHD had one or more. Microhemorrhages were observed in 24 (54.5%) ACHD. Brain volumes did not differ between ACHD with and without lesions (B = −11.49, 95% CI: −97.85; 74.87, *β* = −0.04*, P* = 0.79).

Brain volumes stratified by group are presented in Table 2. TBV, total white matter, cerebellar white matter and corpus callosum were significantly smaller in ACHD than in controls. After adjusting for TBV, only corpus callosum remained significant (Effect of group adjusted for TBV: total white matter: *P* = 0.08; cerebellar white matter: *P* = 0.11; corpus callosum: *P* = 0.03).

**Table 2:** Brain volumes of ACHD vs Controls, cm^3^

Mean (SD)	ACHD, *N* = 44	Controls, *N* = 53	% Difference^a^	*P*^b^
Total brain volume	1067.26 (113.53)	1113.04 (97.88)	−4.11	0.006^c^
Cerebellar white matter	26.99 (3.47)	28.69 (3.49)	−5.93	0.02
Total white matter	432.62 (59.81)	461.75 (52.63)	−6.31	0.001^c^
Corpus callosum	2.86 (0.53)	3.17 (4.49)	−9.88	<0.001^c,d^
Total gray volume	608.71 (59.31)	623.79 (52.58)	−2.42	0.08
Cerebrospinal fluid	23.06 (11.84)	21.86 (9.89)	5.50	0.72

^a^Percentage difference = (mean volume of controls – mean volume of ACHD)/(mean volume of controls) × 100; ^b^Group comparison separately calculated with linear regression with age and sex as covariates, individual brain volume as dependent and group as independent variable, not adjusted for total brain volume, 94 degrees of freedom, *P*-values refer to the predictor group; ^c^Overall model was significant after fdr-correction (all fdr-corrected *P*-values <.05); ^d^Regression model significant after adjusting for total brain volume (F(4, 92) = 21.12, *P* < 0.0001, fdr-adjusted *P* < 0.0001), Effect of group: B = 104, CI: −12.00; −9.26, *β* = −0.17*, P =* 0.03); CI: confidence interval

### Brain imaging and medical factors in ACHD

There was no influence of CPB surgery, cyanosis and complexity of heart defect on TBV (CPB: B = −19.09, 95% CI: −91.61; 53.42, *β* = −0.08, *P* = 0.60; cyanosis: B = −25.21, 95% CI: −93.88; 43.46, *β* = −0.10, *P* = 0.46; complexity of heart defect: moderate vs mild: B = −15.08, 95% CI: −87.61; 57.45, *β* = −0.10, *P* = 0.68; severe vs mild: B = −29.03, 95% CI: −117.68; 59.62, *β* = −0.13, *P* = 0.51).

### Brain imaging and neurocognitive function

**Fig. 1:**
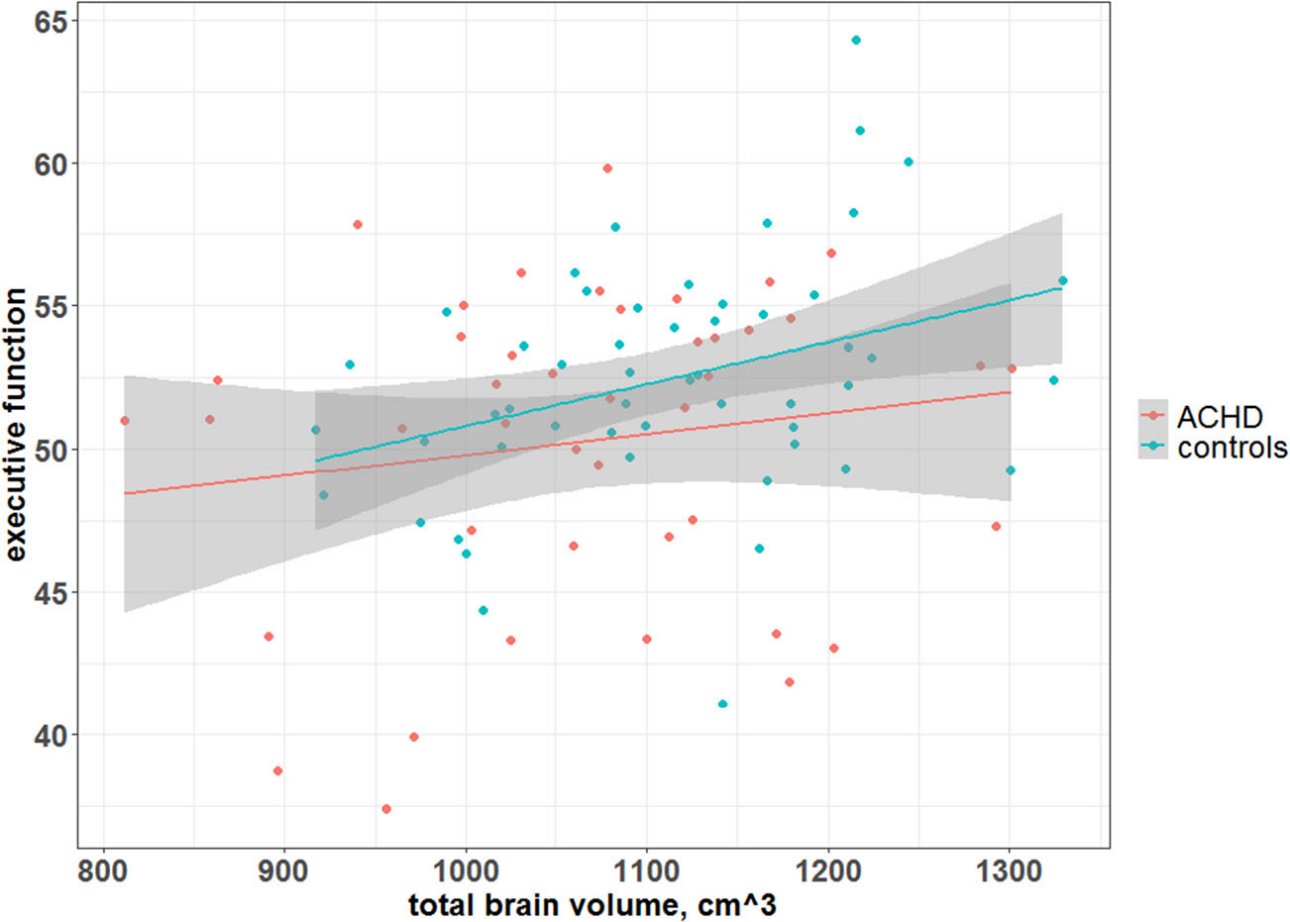
Association of total brain volume with executive function. The linear association of the executive function global score and total brain volume stratified by group (ACHD vs controls) is illustrated. The regression lines have been fitted using linear regression. TBV is positively associated with EF (*P* = 0.02), but there was no interaction of group with TBV on EF (*P* = 0.34)

As illustrated in Fig. 1 and Table 3, TBV and corpus callosum were not associated with IQ (TBV: B = 0.02, 95% CI: −0.02; 0.05), *β* = 0.14, *P* = 0.40; corpus callosum: B = 1.11, 95% CI: −4.41; 6.63, *β* = 0.69 *P =* 0.69). In contrast, TBV was associated with EF (*P* = 0.02), while corpus callosum was not (*P* = 0.80). There was no interaction of group with TBV on EF (interaction: *P* = 0.34). When investigating the association of TBV with EF subdomains (inhibition, working memory, flexibility, planning and fluency) TBV was associated only with inhibition (*P* = 0.008). Interestingly, corpus callosum was also associated with inhibition (*P* = 0.007).

**Table 3:** Association of total brain volume with executive function

	EF global score				Inhibition			
	B	95% CI	β	*P*	B	95% CI	β	*P*
Total brain volume								
Group	8.20	−11.35; 27.74	0.84	0.41	18.92	−8.54; 44.85	1.39	0.18
TBV	0.02	0.00; 0.03	0.40	0.02	0.03	0.00; 0.05	0.44	0.008
TBV*group	−0.01	−0.03; 0.01	−0.96	0.34	−0.02	−0.04; 0.01	−1.47	0.15
	F (5, 89) = 3.63, *P* = 0.004, *fdr-adj P =* 0.015, adj R^2^ = 0.12				F (5, 91) = 2.902, *P* = 0.0178, *fdr-adj P* = 0.031, adj R^2^ = 0.09			
Corpus callosum								
Group	1.36	−10.63; 13.35	0.14	0.82	11.54	−4.62; 27.71	0.88	0.159
CC	0.43	−2.86; 3.71	0.05	0.80	5.30	1.46; 9.13	0.41	0.007
CC*group	−0.86	−4.79; 3.07	−0.26	0.67	−4.20	−9.50; 1.09	−0.94	0.119
	F (6, 88) = 2.85, *P* = 0.014*, fdr-adj P =* 0.027, adj R^2^ = 0.11^a^				F (5, 91) = 2.924, *P* = 0.017, *fdr-adj P* = 0.031, adj R^2^ = 0.091			

^a^adjusted forTBV; B: unstandardized regression coefficients, CI: confidence interval, β: standardized regression coefficients, EF: executive function., TBV: total brain volume, CC: corpus callosum

### Socioeconomic status, brain imaging and executive function

**Fig. 2:**
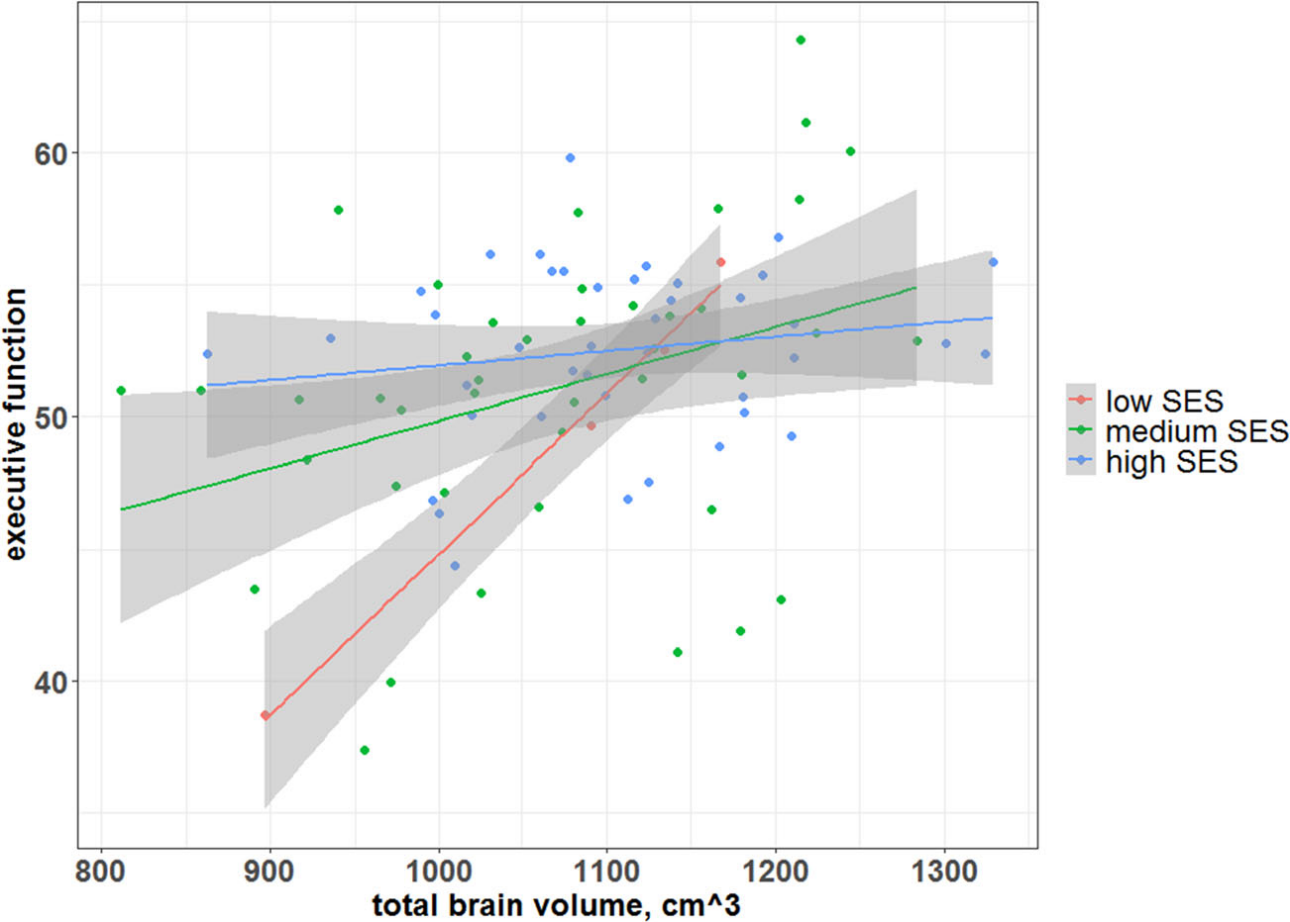
Interaction of SES with total brain volume on executive function in the combined cohort. The linear association of TBV and SES on the executive function global score. SES was stratified (low: 2–5, medium: 6–8, high: 9–12). There was an interaction of SES with TBV on the EF global score for all subjects (*P* = 0.03). In participants with high socioeconomic background, SES buffers the effect of a TBV on EF global score

In a model with SES category and an interaction of group with SES, parental SES was not associated with TBV (B = 14.80, 95% CI: −50.05; 79.66, *β* = 0.16, *P* = 0.65). There was an interaction of SES with TBV on EF in all subjects (F (5, 81) = 5.49, *P <* 0.001, fdr-corrected *P <* 0.001): lower SES category was associated with a stronger effect of TBV on EF than higher SES (B = −0.017, 95% CI: −0.032; −0.002, *β* = −-2.38, *P* = 0.03, see also Fig. 2). When calculating the interaction of SES with TBV on EF for ACHD and controls separately, the interaction was not significant in either group (ACHD: B = −0.017, 95% CI: −4.25; 0.008, *β* = −2.25, *P* = 0.17, controls: B = −0.02, 95% CI: −0.04; 0.001, *β* = −3.19, *P* = 0.06).

## Discussion

In a current cohort of young adults with CHD, we observed smaller global brain volumes compared to controls, which were associated with poorer EF scores but not with lower IQ across all participants. The association between brain volumes and EF was not specific to ACHD, but could also be found in controls. Our results provide evidence that reductions in brain volume described previously for CHD children and adolescents with CHD persist into adulthood and are related to EF abilities.

Smaller global brain volumes in the CHD population are consistently reported for fetuses, neonates, children and adolescents (Limperopoulos et al. 2010; Heinrichs et al. 2014; von Rhein et al. 2014; von Rhein et al. 2015; Bolduc et al. 2018; Heye et al. 2018). We provide evidence that brain volume reductions persist into adulthood even in a cohort of ACHD with normal intellectual functioning. Our findings are consistent with those from a small study in 10 ACHD with chronic cyanosis (Cordina et al. 2014), where the authors described smaller global gray and white matter volumes (Cordina et al. 2014). In our cohort, the white matter seems to be particularly affected, which may be explained by differences in medical risk factors.

The TBV difference between ACHD and controls in our study was 4.1% as opposed to a study by Fontes et al. with a difference of 8.8% (Fontes et al. 2019). In their study, all participants had undergone CPB surgery within the first 2 years of life and thus constitute a population with more severe CHD. Similarly to our study, Fontes et al. found smaller total white matter volume, whereas total gray matter volume was not significantly different.

The white matter seems to be particularly affected in the CHD population, with white matter injuries (WMI) frequently described in neonates (Guo et al. 2018). Neonatal WMI has been associated with impaired perioperative brain growth affecting white and gray matter (Peyvandi et al. 2018). In our cohort of ACHD, brain lesions were not associated with smaller brain volume, however, the full burden of neonatal WMI may not be detectable in adulthood.

Of note, the corpus callosum was the only structure that was smaller in our ACHD after controlling for TBV. This finding has been previously described for adolescents with CHD (Bolduc et al. 2018). A study in neonates with CHD found microstructural changes and decreased volume in the corpus callosum (Hagmann et al. 2016) and suggested a link between bypass surgery and the changes in the corpus callosum. The corpus callosum is crucial for cognitive performance and integration of somatosensory information. (Fryer et al. 2008; Hagmann et al. 2016). A decreased callosal volume in CHD neonates may suggest early dysmaturation with less connectivity, and neonates may not achieve the growth level typical of the general population (Hagmann et al. 2016). Furthermore, a decreased callosal volume may be an indicator of overall less connectivity between the two hemispheres and may persist throughout the lifespan in the CHD population.

In our ACHD and controls, global brain volumes were associated with EF scores. The association between brain volume and cognitive function was previously described in studies in younger CHD populations aged 14 to 22 years (Heinrichs et al. 2014; von Rhein et al. 2014; Semmel et al. 2018). In these studies all participants underwent CPB bypass surgery, while in our cohort only 69% of all ACHD underwent bypass surgery. Our findings may indicate that even a milder form of CHD might affect the development of higher order cognitive abilities like EF. Contrary to a study in adolescents (von Rhein et al. 2014), in our subjects, brain volumes were not associated with IQ. This may indicate that EF are particularly susceptible to the cumulative effect of atypical neurodevelopment, extending into adulthood.

Despite normal IQ in adulthood, our ACHD population had brain abnormalities, such as microhemorrhage and smaller brain volumes on MR imaging. The cumulative burden of brain abnormalities in the CHD population may indicate an overall increased brain vulnerability. This brain vulnerability in combination with cardiovascular comorbidities, such as low cardiac output and coronary disease has been suggested to pose a potential risk for abnormal brain aging and early dementia in the ACHD population (Marelli et al. 2016). A systematic review in ACHD found an increased burden of cerebrovascular damage and cerebral vessel disease supporting the notion of early brain aging in this population (Melazzini et al. 2019). The presence of clinically silent brain abnormalities on MR imaging should warrant clinicians to follow-up CHD patients during adulthood as this patient population may be at increased risk for neurocognitive impairments.

We could not identify any medical risk factors associated with TBV. Neither complexity of heart defect, cyanotic heart defect, nor CPB surgery were associated with TBV in the present dataset. In contrast, in a study in adolescents, a cyanotic heart defect was associated with smaller global brain volume (von Rhein et al. 2014), but the effect of medical factors on global brain volume may vary across ages and diminish across the lifespan. Further, compensatory mechanisms and environmental factors may influence brain volume and neurocognitive outcome. The underlying mechanism leading to altered brain growth is multifactorial, and may consist of multiple hits (Peyvandi et al. 2019). A complex interplay of risk factors, including altered fetal hemodynamics, medical risk factors related to the type of CHD, genetics as well as environmental factors play an important role in the brain development in this at risk population. Longitudinal study designs are necessary to further understand the interplay of risk/protective factors on neurodevelopmental trajectory.

Of note, we found a negative interaction of parental SES with total brain volume on executive functioning for all subjects. This suggests that for subjects with a higher SES, the brain volume has little effect on neurocognitive functioning, while the association of brain volumes with executive functions is stronger for subjects with a low SES. Similar results were previously reported in children with CHD and in another at risk population of preterm children (Benavente-Fernández et al. 2019; Naef et al. 2019). SES among other factors may contribute to compensatory mechanisms and may explain why ACHD in our study showed normal cognitive function despite having smaller global brain volumes.

Our results suggest that the importance of medical risk factors for long-term outcome in adults with CHD is minor compared to environmental factors (e.g. SES).

This is a single-centered study with patients coming from high socioeconomic background and cognitive outcome within the norm and not all patients underwent CPB surgery. Despite our ACHD population being a high functioning and rather healthy one, we found global brain volumes to be smaller than in healthy peers. We did not find any difference between heard defect severity and global brain volume, however as numbers for a specific heart defect were small, we could not analyse the influence of specific defects on brain volume in more detail.

Brain alterations may be present in the ACHD population, irrespective of cardiac defect. These alterations are associated with mild executive dysfunctions. Brain volume changes are possibly due to impaired growth in the white matter. The implications of these findings in relation to early brain aging are yet unclear. However, our findings emphasize that health care professionals should provide comprehensive follow-up programs for adults with CHD as suggested by Kovacs and Bellinger (Kovacs and Bellinger 2020), particularly for those coming from low socioeconomic background, thereby providing opportunities for social and educational support to foster cognitive development.

## Supplementary Information

ESM 1 (DOCX 284 kb)

ESM 2 (DOCX 48 kb)

## Data Availability

The de-identified data are available from the corresponding author upon reasonable request.

## Abbreviations

ACHD: Adults with congenital heart disease
CHD: Congenital heart disease
CPB: Cardiopulmonary bypass
EF: Executive function
IQ: Intelligence quotient
IQR: Interquartile range
MRI: Magnetic resonance imaging
SD: Standard deviation
SES: Socioeconomic status
TBV: Total brain volume
WMI: White matter injury
