# Supplementary material for: Brain volumes in adults with congenital heart disease correlate with executive function abilities
Source: Brain Imaging Behav. 2021 Jan 30;15(5):2308–16. doi: 10.1007/s11682-020-00424-1 (PMC8500877; doi:10.1007/s11682-020-00424-1)
Supplement: Supplementary file 1 — (DOCX 284 kb) [file 11682_2020_424_MOESM1_ESM.docx]

Supplemental Material: Description of MRI processing and imaging sequences used to evaluate focal and multifocal brain abnormalities


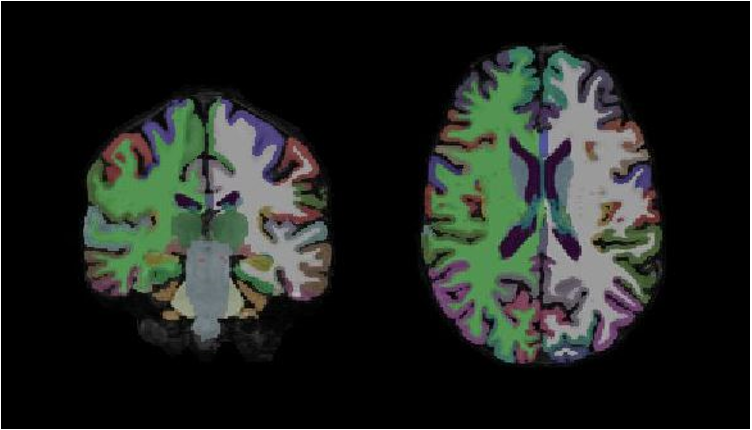


Supplemental Figure 3: Volumetric segmentation of MR images of one participant

Illustration of volumetric segmentation calculated using Freesurfer. Axial and frontal view of a T1 slice. Total white matter (right: green, left: white) and cortical gray matter (red) are colored in the T1 image of one study participant.

The processing of MR images included removal of non-brain tissue using a hybrid watershed/surface deformation procedure (Ségonne *et al.*, 2004), automated Talairach transformation, segmentation of the subcortical white matter and deep gray matter volumetric structures (Fischl *et al.*, 2002; Fischl *et al.*, 2004; Ségonne *et al.*, 2004) intensity normalization (Sled *et al.*, 1998), tessellation of the gray matter/white matter boundary, automated topology correction (Fischl *et al.*, 2001; Ségonne *et al.*, 2007), and surface deformation following intensity gradients to optimally place the gray/white and gray/cerebrospinal fluid borders at the location where the greatest shift in intensity defines the transition to the other tissue class (Dale and Sereno, 1993; Dale *et al.*, 1999; Fischl and Dale, 2000). This method uses both intensity and continuity information from the entire 3D MR volume in segmentation and deformation procedures to produce representations of cortical thickness, calculated as the closest distance from the gray/white boundary to the gray/CSF boundary at each vertex on the tessellated surface (Fischl and Dale, 2000). The following sequences were used to evaluate brain abnormalities: SPGR: T1 weighted three dimensional spoiled gradient echo pulse sequence, T2-weighted sagittal fast spin-echo sequence (repetition time (TR) = 10000ms, echo time (TE) = 95ms, field of view (FOV) = 260mm, matrix = 512x512mm, slice thickness (ST) = 3mm), 3D T2-weighted fast spin-echo sequence (TR = 2800ms, TE = 95ms, FOV = 256mm, matrix = 256x256mm, ST = 1mm), SWI: Susceptibility-weighted sequence (SWI) (TR = 53ms, TE = 31ms, FOV = 240mm, matrix = 512x 512mm, ST = 3mm) and DTI: Diffusion tensor imaging (TR = 6500ms, TE = 85ms, FOV = 280mm, matrix 256x256 mm, ST = 3.6mm).

References:

Dale AM, Fischl B, Sereno MI. Cortical surface-based analysis. I. Segmentation and surface reconstruction. Neuroimage 1999; 9(2): 179-94.

Dale AM, Sereno MI. Improved Localizadon of Cortical Activity by Combining EEG and MEG with MRI Cortical Surface Reconstruction: A Linear Approach. J Cogn Neurosci 1993; 5(2): 162-76.

Fischl B, Dale AM. Measuring the thickness of the human cerebral cortex from magnetic resonance images. Proc Natl Acad Sci U S A 2000; 97(20): 11050-5.

Fischl B, Liu A, Dale AM. Automated manifold surgery: constructing geometrically accurate and topologically correct models of the human cerebral cortex. IEEE Trans Med Imaging 2001; 20(1): 70-80.

Fischl B, Salat DH, Busa E, Albert M, Dieterich M, Haselgrove C*, et al.* Whole brain segmentation: automated labeling of neuroanatomical structures in the human brain. Neuron 2002; 33(3): 341-55.

Fischl B, Salat DH, van der Kouwe AJ, Makris N, Ségonne F, Quinn BT*, et al.* Sequence-independent segmentation of magnetic resonance images. Neuroimage 2004; 23 Suppl 1: S69-84.

Sled JG, Zijdenbos AP, Evans AC. A nonparametric method for automatic correction of intensity nonuniformity in MRI data. IEEE Trans Med Imaging 1998; 17(1): 87-97.

Ségonne F, Dale AM, Busa E, Glessner M, Salat D, Hahn HK*, et al.* A hybrid approach to the skull stripping problem in MRI. Neuroimage 2004; 22(3): 1060-75.

Ségonne F, Pacheco J, Fischl B. Geometrically accurate topology-correction of cortical surfaces using nonseparating loops. IEEE Trans Med Imaging 2007; 26(4): 518-29.
