# Supplementary material for: Brain volumes in adults with congenital heart disease correlate with executive function abilities
Source: Brain Imaging Behav. 2021 Jan 30;15(5):2308–16. doi: 10.1007/s11682-020-00424-1 (PMC8500877; doi:10.1007/s11682-020-00424-1)
Supplement: Supplementary file 2 — (DOCX 48 kb) [file 11682_2020_424_MOESM2_ESM.docx]

Supplemental Table 4: Cardiac diagnosis stratified by mild, moderate and severe:

|  | N (%) |
| --- | --- |
| Mild CHD | 15 (33.3) |
| Isolated congenital aortic valve disease | 6 (13.3) |
| Ventricular septal defect - repaired | 2 (4.4) |
| Isolated congenital mitral valve disease | 2 (4.4) |
| Previously ligated or occluded ductus arteriosus | 1 (2.2) |
| Repaired ventricular septal defect with tricuspid valve disease | 1 (2.2) |
| Mild pulmonary stenosis | 1 (2.2) |
| Small atrial septal defect | 1 (2.2) |
| Congenital mitral valve disease and small atrial septal defect | 1 (2.2) |
| Moderate CHD | 20 (45.5) |
| Coarctation of the aorta | 6 (13.3) |
| Tetralogy of Fallot | 3 (6.7) |
| Subvalvar aortic stenosis | 1 (2.2) |
| Ebstein anomaly | 2 (4.4) |
| Ventricular septal defect - with coarctation of the aorta | 3 (6.7) |
| Ventricular septal defect - with right ventricular outflow tract obstruction | 2 (4.4) |
| Ventricular septal defect - with mitral disease | 2 (4.4) |
| Abnormal origin of the left pulmonary artery and ventricular septal defect | 1 (2.2) |
| Severe CHD | 9 (20.5) |
| Transposition of the great arteries^a^ | 7 (15.6) |
| Fontan procedure | 2 (4.4) |

^a^atrial switch (Senning/Mustard), N=5 (11.1)
